# Studies on the alterations in haematological indices, micronuclei induction and pathological marker enzyme activities in *Channa punctatus* (spotted snakehead) perciformes, channidae exposed to thermal power plant effluent

**DOI:** 10.1186/s40064-016-2478-9

**Published:** 2016-06-17

**Authors:** Mehjbeen Javed, Irshad Ahmad, Ajaz Ahmad, Nazura Usmani, Masood Ahmad

**Affiliations:** Aquatic Toxicology Research Laboratory, Department of Zoology, Aligarh Muslim University, Aligarh, Uttar Pradesh 202002 India; Department of Biochemistry, Faculty of Life Sciences, Aligarh Muslim University, Aligarh, 202002 Uttar Pradesh India

**Keywords:** Heavy metals, Haematology, Genotoxicity, Transferase, Phosphatase, Kinase

## Abstract

The present study was conducted to assess the toxicity of thermal power plant effluent containing heavy metals (Fe > Cu > Zn > Mn > Ni > Co > Cr) on haematological indices, micronuclei, lobed nuclei and activity of pathological marker enzymes [alkaline phosphatase (ALP), aspartate transferase (AST), alanine transferase (ALT) and creatine kinase (CK)] in *Channa punctatus*. Total erythrocyte count (−54.52 %), hemoglobin (−36.98 %), packed cell volume (−36.25 %), mean corpuscular hemoglobin concentration (−1.41 %) and oxygen (O_2_) carrying capacity (−37.04 %) declined significantly over reference fish, however total leukocyte count (+25.43 %), mean corpuscular hemoglobin (+33.52 %) and mean corpuscular volume (+35.49 %) showed elevation. High frequency of micronuclei (1133.3 %) and lobed nuclei (150 %) were observed in exposed fish which may indicate mutagenesis. Activities of pathological marker enzymes ALP, AST, ALT and CK increased significantly in serum of exposed fish. The ratio of ALT: AST in exposed fish was beyond 1 which indicates manifestation of pathological processes. These biomarkers show that fish have macrocytic hypochromic anemia. Leukocytosis showed general defence response against heavy metal toxicity and marker enzymes showed tissue degeneration. In conclusion, thermal power plant effluent has strong potential to induce micronuclei, tissue pathology, making the fish anemic, weak, stressed and vulnerable to diseases.

## Background

Industrial activities results into the production of huge waste which is usually discharged into the nearby water bodies leading to the pollution of aquatic ecosystems. Heavy metals are persistent and accumulative in nature therefore could pose detrimental effects to the inhabiting flora and fauna. Fishes are widely employed as biomonitoring organisms in toxicological studies as they can highlight the potential dangers of toxicants introduced in the aquatic environment (Crafford and Avenant-Oldewage 2010; Javed and Usmani 2011, 2013a, b, c; Javed 2013; Ahmad and Ahmad 2015; Javed et al. 2016). Uptake of heavy metals occurs through the fish gills from water and gut via food then they pass to the blood stream and finally settled to different organs (Javed and Usmani 2012). Therefore a number of haematological indices such as haematocrit (Hct), haemoglobin (Hb), total erythrocyte (TEC) and total leucocyte counts (TLC), mean corpuscular haemoglobin (MCH) and oxygen carrying capacity (O_2_ C.C) are used to assess the health status of the bioindicator and environmental pollution (Shah and Altindag 2005; Javed and Usmani 2015). Frequency of micronuclei induction has also been useful to assess the mutagenic potential of the wastewaters. Micronucleus test detects both clastogenic and aneugenic effects of the toxicants therefore it is widely used to detect the genotoxicity of pollutants (Heddle et al. 1991). Aspartate aminotransferase (AST), alanine aminotransferase (ALT), alkaline phosphatase (ALP) and creatine kinase (CK) are important pathological marker enzymes. Increase or decrease in their activities gives an indication of tissue injury, environmental stress or diseased condition (Kori-Siakpere et al. 2012). In mammals blood parameters are widely used to assess the alteration in physiology. In fishes also they serve as useful biomarkers to assess the in vivo environmental exposures (Zutshi et al. 2010; Hanan et al. 2013; Javed and Usmani 2012). Therefore the aim of the present study is the evaluation of haematoxic and genotoxic potential of heavy metal loaded thermal power plant effluent in fish *Channa punctatus*.

## Methods

### Sample collection

Water sample was collected in acid rinsed bottles for estimation of heavy metals. Heavy metals in water were estimated as per standard methods given in APHA (2005).

Live samples of fish *C. punctatus* (n = 10), (12.5 ± 1.1 cm; 43.6 ± 1.4 g) were collected from the canal receiving thermal power plant effluent. Healthy reference fishes (n = 10) (15 ± 2.1 cm: 50.0 ± 1.9 g) were also procured from the nearby Sumera reservoir (20.933° N and 77.333 E), district Aligarh, India. It has no known source of pollution. All the fishes were collected by the help of cast net and local fisherman. They were transported to laboratory individually for further studies. The experiment was approved by the animal ethical committee at the Department of Zoology, A.M.U, Aligarh.

### Blood count parameters and oxygen carrying capacity

Prior to collection of blood, fishes were hit by a blow on head to dislocate cervical. Blood was collected by cardiac puncture using disposable syringes and kept in separate vials (with EDTA and without anticoagulant). All the blood samples were handled at room temperature. EDTA containing blood was used for whole blood count parameters. Total erythrocyte count (TEC) (10^6^ mm^−3^) and leucocyte count (TLC) (10^3^ mm^−3^) were quantified by neubauer hemocytometer (Rohem, India). Haemoglobin concentration (Hb) (g dL^−1^) was determined with haemoglobin test kit (DIAGNOVA, Ranbaxy, India). Packed cell volume (PCV) (%) was estimated by Wintrobes tube method. Mean corpuscular haemoglobin (MCH), Mean corpuscular haemoglobin concentration (MCHC) and Mean corpuscular volume (MCV) were calculated using the following formulae:

$${\text{MCH}}\;\left( {\text{pg}} \right) = \frac{{{\text{Hb}}\left( {\text{g/dL}} \right) \times 10}}{{{\text{TEC}}\;\left( {10^{6} \;{\text{mm}}^{ - 3} } \right)}}$$

$${\text{MCHC}}\;\left( {{\text{g}}\;{\text{dL}}^{ - 1} } \right) = \frac{{{\text{Hb}}\;\left( {\text{g/dL}} \right) \times 100}}{{{\text{PCV}}\,\left( \% \right)}}$$

$${\text{MCV}}\;\left( {\upmu{\text{m}}^{3} } \right) = \frac{{{\text{PCV}}\,( \% ) \times 10}}{{{\text{TEC}}\,\left( {10^{6} \;{\text{mm}}^{ - 3} } \right)}}$$

Oxygen carrying capacity (O_2_C.C) is obtained by multiplying the Hb content with the O_2_ combining power of 1.25 ml of O_2_ per g Hb (Johansen 1970).

### Micronuclei test (MNT)

Blood smear of reference and exposed fishes were prepared to observe micronuclei (MN) and other morphological changes induced in blood cells. Smear was fixed with 100 % methanol, air dried and then stained with 10 % giemsa solution for 10 min. Slides were air dried overnight, mounted and then micro-nucleated cells were scored. From each animal, 1000 erythrocytes were scored to determine the frequency of micronuclei. Only the cells clearly isolated from the surrounding cells were scored. The criteria for the identification of micronuclei were as follows: (a) MN must be smaller than one-third of the main nuclei, (b) MN must be clearly separated from the main nuclei, (c) MN must be on the same plane of focus and have the same color. Cells having two or more nuclei with approximately equal sizes were considered as bi-nucleated and multinucleated cells (Das and Nanda 1986).

### Pathological marker enzyme activity in serum

The non EDTA blood was let to stand at room temperature, centrifuged at 3000 rpm for 10 min to obtain serum.

Aspartate aminotransferase (AST) was assayed using the diagnostic kit (Span Cogent Diagnostics, India). Alanine aminotransferase (ALT) activity was quantitated using the Techno Biomed diagnostic kit (Jupiter reagents, New Delhi, India). Alkaline phosphatase (ALP) was measured using the diagnostic kit (Cogent, span diagnostics Ltd. India) and Creatine kinase (CK) using the kit Infinite CK-NAC (Accurex biomedical Pvt. Ltd., India).

### Statistical analysis

Analysis of all these parameters was done in duplicates. All values are given as mean ± SEM (standard error of mean). Data has been statistically analyzed with the help of Student’s t test using software SPSS, version 16.

## Results

In Kasimpur canal water heavy metals were estimated in the order Fe (8.7 mg/L) > Cu (0.86 mg/L) > Zn (0.3 mg/L) > Mn (0.2 mg/L) > Ni (0.12 mg/L) > Co (0.11 mg/L) > Cr (0.1 mg/L). In the reference reservoir Fe (0.2 mg/L) and Cu (0.04 mg/L) were present while others were below the detection limit.

Table 1 summarizes the effect of heavy metals on haematological indices, micronuclei and enzyme activities of *C. punctatus*. TEC, Hb and PCV decline significantly (p < 0.001) as compared to reference fish. In contrast, TLC showed significant (p < 0.05) elevation over reference fish. Both MCH and MCV showed significant (p < 0.05) rise but MCHC drop significantly (p < 0.001) on comparison to the reference. Similarly O_2_ C.C. showed significant (p < 0.001) decrease.

**Table 1:** Impact of thermal power plant effluent on haematological indices, micronuclei and enzyme activities of *Channa punctatus*

Variables	Reference fish	Exposed fish	% Change over reference
TEC (10^6^ mm^−3^)	5.74 ± 0.55	2.61 ± 0.21***	−54.52
TLC (10^3^ mm^−3^)	4.56 ± 0.16	5.72 ± 1.23*	+25.43
Hb (g dL^−1^)	13.06 ± 0.12	8.23 ± 0.37***	−36.98
PCV (%)	40.18 ± 0.36	25.69 ± 1.12***	−36.25
MCH (pg)	23.77 ± 2.72	31.74 ± 1.39*	+33.52
MCHC (g dL^−1^)	32.49 ± 0.01	32.03 ± 0.06***	−1.41
MCV (µm^3^)	73.14 ± 6.30	99.1 ± 4.49*	+35.49
O_2_ C.C (Vol%)	16.33 ± 0.32	10.28 ± 0.46***	−37.04
Micronuclei	0.06 ± 0.001	0.74 ± 0.003***	+1133.3
Lobed Nuclei	0.02 ± 0.001	0.05 ± 0.001	+150
ALT	4.30 ± 1.0	32.40 ± 0.02***	+653.48
AST	9.18 ± 1.00	31.0 ± 1.00	+237.69
ALT: AST	0.47 ± 0.05	1.04 ± 0.03**	+121.27
ALP	0.89 ± 0.01	1.53 ± 0.20***	+71.91
CK	94.1 ± 0.10	280.0 ± 0.0***	+197.55

Values are given as mean ± SEM, (n = 10), Exposed and reference values are replicates of 10; Level of significance established at * p < 0.05, ** p < 0.01; *** p < 0.001; + or – sign indicates increase or decrease over reference values

**Fig. 1:**
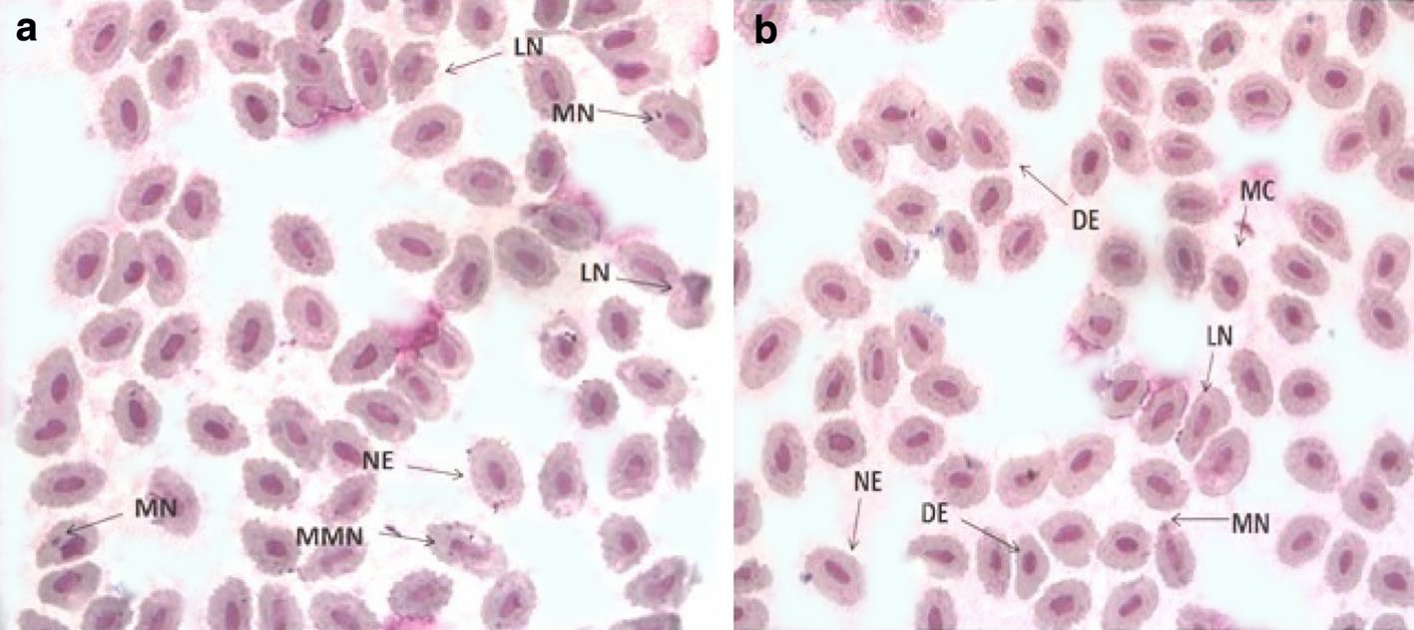
**a**, **b** Illustrates the alteration in blood cells of exposed *C. punctatus*. MN (micronucleus), LN (lobed nucleus), NE (normal erythrocyte), MMN (multiple micronuclei), DE (distorted erythrocyte), MC (microcyte)

In erythrocytes frequency of micronucleated cells were also significantly (p < 0.001) higher relative to reference fish (Table 1). In addition to micronuclei some other abnormalities were also observed such as lobed nucleus, distorted erythrocytes, and few microcytes (Fig. 1).

Similarly activities of all the studied enzymes namely ALT, AST, ALP and CK were all elevated as compared to the reference fish. ALT: AST ratio was higher than 1 in exposed fish.

## Discussion

Heavy metals in Kasimpur canal water were present in the following order: Fe > Cu > Zn > Mn > Ni > Co > Cr where Fe and Ni content exceeded the recommended guidelines of both Bureau of Indian standards (BIS 1992) and United Nations Environment Programme Global Environment Monitoring System (UNEPGEMS 2006). Whereas in the reference reservoir located at Sumera, district Aligarh only Fe and Cu were detected which were within the permissible limits.

Study on whole blood count, micronuclei induction and biochemical changes in fish serves as an effective tool in the diagnosis of stress, mutagenesis, environmental pollution and also the abiotic fish diseases as changes in blood appears first before the onset of any morphological or degenerative changes. The haematological indices which are measured in the present study are a part of complete blood count (CBC). TEC, Hb and TLC are often used in the general evaluation of animal’s health. Changes in these indices from reference give an indication of disease. Low TEC usually leads to low PCV and Hb levels, which has also been observed in the current study. Shaheen and Akhtar (2012) also reported significant decline in Hb content and TEC counts of fish *Cyprinus carpio* when exposed to Cr(VI). Decrease in TEC, Hb and PCV indicates anemic condition of the animal. This anemic condition could be due to the damage of haemopoietic tissue or inhibition of erythropoiesis and transferrin dysfunction. MCV, MCH and MCHC are the part of full blood count and they are also called as red cell indices. It means that they express the size and haemoglobin content of erythrocytes. MCV defines the size of the erythrocytes where high MCV indicates macrocytic condition and low as microcytic condition. Therefore in the present case, heavy metal exposure induced macrocytic anemia in the fish since MCV showed higher levels as compared to the reference fish. This is a critical condition that occurs when cell fails in producing DNA quickly enough to divide at the right moment because it grows continuously to larger sizes (Hoffman et al. 2009; Wang et al. 2009; Braunwald et al. 2001). MCH was higher and MCHC lowered significantly in exposed *C. punctatus*. Similar trend decline in PCV, increase in MCV and MCH and drop in MCHC levels have also been reported in fish *L. rohita* when exposed to effluents of paint, dye and petroleum industry (Zutshi et al. 2010). Shalaby (2001) reported significant rise in MCV and decrease in MCH and MCHC values in *Oreochromis mossambicus* when exposed to heavy metal Cd. MCH and MCHC both describes the average haemoglobin content of erythrocytes but in a different way. MCH expresses content of haemoglobin per red cell whereas MCHC reflects amount of haemoglobin per unit volume of red cell. Low MCHC values indicates hypochromic anemia. According to Hoffman et al. (2009), Wang et al. (2009) and Braunwald et al. (2001) macrocytic hypochromic anemia occurs due to arrest in nuclear maturation of erythrocytes relative to cytoplasmic maturity and these abnormalities are due to impaired DNA synthesis. These megaloblastic changes are most apparent in rapidly dividing cells such as blood cells (Hoffman et al. 2009; Wang et al. 2009; Braunwald et al. 2001). In the current study leukocytosis was observed in exposed fishes. Other workers have also reported similar observation in *C. punctatus* exposed to Pb (Hymavathi and Rao 2000), *Clarias batrachus* exposed to HgCl_2_ (Joshi et al. 2002), *C. carpio* exposed to Cr(VI) (Shaheen and Akhtar 2012). Hanan et al. (2013) reported much higher values of leukocyte count in fish *C*. *gariepinus* inhabiting El-Rahawy delta of River Nile which receive industrial, domestic and agricultural waste. Increase in leukocytes in all these cases including the present one could be attributed to their role in defense. And also, leukocytosis is directly proportional to the severity of damage and stress induced by heavy metals which as a consequence result in the stimulation of immunological defense (Javed and Usmani 2012). According to Hanan et al. (2013), the relative decrease/increase in hematological indices had proved the toxic effects of heavy metals that influence both metabolic and hemopoietic activities in the animal. In the present study O_2_C.C decline significantly since it depends on Hb of erythrocytes. When fish are exposed to heavy metals they bind with Hb replacing the oxygen. Other workers have also reported the decrease of O_2_C.C in fishes such as *H*. *fossilis* exposed to mixture of Cu and NH_3_ (James and Sampath 1995), *O. mossambicus* exposed to Cu and Zn (Sampath et al. 1998). This decline could be due to the fact that heavy metals damage the structure of RBC hence instead of four, less molecules of oxygen bind to the hemoglobin. The loss of hemoglobin and consequent reduction in the O_2_C.C of the blood is the most conspicuous feature of anemia.

Micronuclei are the small part of chromosomes left during anaphase stage of cell division due to some abnormality during their migration to opposite poles. In the study undertaken high percentage (1133.3 %) of micronuclei induction was observed in erythrocytes over the reference fish. Other investigators also reported micronuclei induction in fish *Oreochromis niloticus*, *Anguilla anguilla* as a result of exposure to refinery effluent and urban wastewater containing heavy metals (Da Silva Souza and Fontanetti 2006; Yildiz et al. 2010). Presence of micronuclei is an irreversible change and reflects mutagenesis. In addition to micronuclei some other abnormalities in erythrocytes observed were lobed nucleus, multiple micronuclei and distorted erythrocytes. This could be due to the reason of impaired DNA synthesis which consequentially leads to macrocytic hypochromic anemia. When toxicant impact is observed during cell division, it can produce a mutation which can be transmitted to future generations leading to aneuploidy, impaired reproduction, low survival and may threaten the species (Valavanidis et al. 2006; Almeida et al. 2007; Hwang and Kim 2007; Monserrat et al. 2007).

In the present study activity of enzymes AST, ALT, ALP and CK were measured since they are used as pathological markers, when any damage occurs to the tissues they are spilled into the blood in high active amounts. These enzymes are found in liver, skeletal muscle, kidney, bone etc. AST and ALT are the most sensitive and widely used liver enzymes and are therefore employed as potential marker enzymes of liver damage. In the present study the activity of AST, ALT were high in serum as compared to the reference. Other workers also reported similar findings for AST and ALT in serum of fishes *C. carpio* exposed to Cd and Cr (De Smet et al. 2000; Parvathi et al. 2011), *Tor tor* (Yousafzai and Shakoori 2011), *O. niloticus* (Cogun and Sahin 2013). Tietz (1987) and Campbell et al. (1984) reported that these enzymes liberate to the blood stream when the hepatic parenchyma cells are damaged. The increased activity of AST and ALT indicates the increased rate of transamination as a result protein breakdown to free amino acids for subsequent utilization in glycogenic pathway. Lynch et al. (1969) have reported that even a slightly moderate elevated level of AST in serum is associated with hepatitis and muscular dystrophy in mammals. In the present study the ALT activity was relatively higher than AST in serum of exposed fish. Therefore the ratio of ALT:AST observed was relatively higher than 1 in serum. According to Tapasya and Kunzang (2007) in sever tissue damage ALT activity is higher than AST and the ALT:AST ratio becomes >1 (normally <1).

Alkaline phosphatase is a membrane bound enzyme found at bile pole of hepatocytes and also found in pinocytic vesicle and Golgi complex. It is present on all cell membranes where active transport occurs, and hydrolase and transphosphorylase in function. It is often employed to access the integrity of plasma membrane (Akanji et al. 1993), since it is localized predominantly in the microvilli of the bile canaliculi, located in the plasma membrane. Elevation of this enzyme found in pathological condition such as liver impairment, kidney dysfunction and bone disease (Kopp and Hetesa 2000; Yang and Chen 2003). Since ALP is a membrane-bound enzyme, therefore exposure to heavy metal causes disruption of tissue membrane and change in their properties could alter the ALP activity.

Similarly CK is also a pathological marker enzyme normally present in high concentration in the cytoplasm of myocytes and in lower concentrations in bones and liver. Its function is to catalyze the conversion of creatine to phosphocreatine by splitting itself in the conversion of ATP. In the present study high enzyme activity was reported in serum than reference. Similar response was reported in *C. carpio* (Luskova et al. 2002). However in other studies decrease was also observed in serum in *C. gariepinus*, exposed to Cr (Kori-Siakpere et al. 2012). Skeletal and cardiac muscle damage results in great increase of plasma creatine phosphokinase (CPK) or CK.

## Conclusion

The results of the present investigation confirms that wastewater of thermal power plant induce micronuclei, cause erythrocyte destruction consequently leading to anemia, elevation in pathological marker enzymes activities due to target tissue injury. It has also been noted that DNA synthesis gets impaired due to mutation in erythrocytes which could lead to developmental deformities in fishes and may threaten the species. Therefore haemotological count, micronuclei induction and enzyme activity can suitably be used as early biomarkers of fish health and *C. punctatus* as a good model of environmental exposure. The canal water is also used for irrigation and drinking purposes, thus it may cause similar detrimental effects in non-target organisms like other animals and humans.
